# Breast ductal lavage for biomarker assessment in high risk women: rationale, design and methodology of a randomized phase II clinical trial with nimesulide, simvastatin and placebo

**DOI:** 10.1186/1471-2407-12-575

**Published:** 2012-12-05

**Authors:** Matteo Lazzeroni, Aliana Guerrieri-Gonzaga, Davide Serrano, Massimiliano Cazzaniga, Serena Mora, Chiara Casadio, Costantino Jemos, Maria Pizzamiglio, Laura Cortesi, Davide Radice, Bernardo Bonanni

**Affiliations:** 1 Division of Cancer Prevention and Genetics, European Institute of Oncology, Via Ripamonti 435, Milan, 20141, Italy; 2 Unit of Diagnostic Cytology, Division of Pathology and Laboratory Medicine, European Institute of Oncology, Milan, Italy; 3 Hospital Pharmacy, European Institute of Oncology, Milan, Italy; 4 Breast Radiology Unit, European Institute of Oncology, Milan, Italy; 5 Department of Oncology & Haematology of the University of Modena and Reggio Emilia, Modena, Italy; 6 Department of Epidemiology and Biostatistics, European Institute of Oncology, Milan, Italy

**Keywords:** Clinical trial, Breast cancer prevention, Ductal lavage, Nimesulide, Simvastatin, Intraepithelial neoplasia, Familial risk

## Abstract

**Background:**

Despite positive results from large phase III clinical trials proved that it is possible to prevent estrogen-responsive breast cancers with selective estrogen receptor modulators and aromatase inhibitors, no significant results have been reached so far to prevent hormone non-responsive tumors. The Ductal Lavage (DL) procedure offers a minimally invasive method to obtain breast epithelial cells from the ductal system for cytopathologic analysis. Several studies with long-term follow-up have shown that women with atypical hyperplasia have an elevated risk of developing breast cancer. The objective of the proposed trial is to assess the efficacy and safety of a daily administration of nimesulide or simvastatin in women at higher risk for breast cancer, focused particularly on hormone non-responsive tumor risk. The primary endpoint is the change in prevalence of atypical cells and cell proliferation (measured by Ki67) in DL or fine needle aspirate samples, after 12 months of treatment and 12 months after treatment cessation.

**Methods-Design:**

From 2005 to 2011, 150 women with a history of estrogen receptor negative ductal intraepithelial neoplasia or lobular intraepithelial neoplasia or atypical hyperplasia, or unaffected subjects carrying a mutation of BRCA1 or with a probability of mutation >10% (according to BRCAPRO) were randomized to receive nimesulide 100mg/day versus simvastatin 20mg/day versus placebo for one year followed by a second year of follow-up.

**Discussion:**

This is the first randomized placebo controlled trial to evaluate the role of DL to study surrogate endpoints biomarkers and the effects of these drugs on breast carcinogenesis. In 2007 the European Medicines Agency limited the use of systemic formulations of nimesulide to 15 days. According to the European Institute of Oncology Ethics Committee communication, we are now performing an even more careful monitoring of the study participants. Preliminary results showed that DL is a feasible procedure, the treatment is well tolerated and the safety blood tests do not show any significant liver toxicity. There is an urgent need to confirm in the clinical setting the potential efficacy of other compounds in contrasting hormone non-responsive breast cancer. This paper is focused on the methodology and operational aspects of the clinical trial.

**Trial Registration:**

(ClinicalTrials.gov Identifier: NCT01500577)

## Background

Breast cancer (BC) is now the most common cancer diagnosed in women worldwide and is the leading cause of deaths from cancer among women [1]. Recently BC prevention has been greatly improved and the chemopreventive efficacy of various compounds, particularly Selective Estrogen Receptor Modulators (SERMs) and more recently aromatase inhibitors (AIs), has been repeatedly documented. However these drugs have shown to be effective almost exclusively in hormone-responsive (ER positive) BCs. At least one-third of BCs will not be influenced by hormonal interventions because of the absence of ER expression since the beginning and another number of cancers will subsequently “escape” the hormonal control and become resistant to tamoxifen and AIs. Unfortunately, ER negativity is frequently combined with other characteristics of biological aggressiveness (high grade and proliferation, overexpression of HER2/neu), resulting in a worse prognosis [2,3]. Furthermore, women with a family history of breast and ovarian cancer have a higher risk of developing ER negative BC compared with the general population. In particular BRCA-1 mutation carriers have approximately 90% ER negative tumours, and display a characteristic gene expression profile [4]. For all these reasons, methods to better select subjects at higher risk for ER negative BC and strategies to prevent it are actively being sought.

Several studies with long-term follow-up have shown that women with atypical hyperplasia have an elevated risk of developing breast cancer [5-8].

The ductal lavage (DL) procedure offers a minimally invasive method to obtain breast epithelial cells from the ductal system for cytopathologic analysis to provide individualized risk assessment with a sensitivity up to 3.2 times greater than that of Nipple Aspirate Fluid (NAF) in detecting abnormal intraductal cells [9].

Over-expression of cyclooxygenase-2 (COX-2) has been detected in a variety of human tumors in breast, prostate, lung, skin, and colon [10]. Nimesulide, a preferential COX-2 inhibitor, has been used clinically as an anti-inflammatory agent in Europe, Asia and Africa. COX-2 inhibition by nimesulide has been shown to inhibit cancer cell proliferation and induce cancer cell apoptosis in vitro [11,12], and prevent tumor growth and metastasis in vivo [13-15]. However, COX-2/PGE2-independent mechanisms have also been reported to mediate the anti-tumor activity of nimesulide [16,17].

Statins (HMG-CoA reductase inhibitors), the most widely used medications in the western world to manage hypercholesterolemia and associated morbidities [18], may affect the occurrence or outcomes of other diseases—including cancer—either by downstream consequences of cholesterol reduction or by mechanisms outside of the cholesterol synthesis pathway [19,20]. A recent metanalisis showed that Simvastatin, a highly lipophilic statin, was associated with a reduced risk of breast cancer recurrence among Danish women diagnosed with stage I–III breast carcinoma, whereas no association between hydrophilic statin use and breast cancer recurrence was observed [21].

All these data, together with the long post-marketing surveillance of both compounds, make these two drugs most interesting to investigate in a chemoprevention trial in subjects at higher risk for ER negative breast cancer.

We are conducting a phase II, randomized, double blind, placebo controlled trial in 150 women at increased risk for hormone non-responsive breast cancer, randomly assigned to receive nimesulide 100 mg or simvastatin 20 mg once daily or matching placebo for 12 months, and then followed for another year. This paper describes the rationale and design of the study, thus focusing on the methodology and operational aspects of the clinical trial.

## Methods

### Ethical considerations and registration

The study protocol is in compliance with the Declaration of Helsinki [22]. We obtained approval for this study from the Ethics committee of the European Institute of Oncology on February 3rd 2005 (EUDRACT N.: 2004-005267-21). The protocol and informed consent forms were approved by the institutional ethics committee at each of the participating institutions. The trial has been registered with the ClinicalTrials.gov Identifier: NCT01500577. Written informed consent for participation in the study has been obtained from all the participating patients.

### Design overview

**Figure 1:**
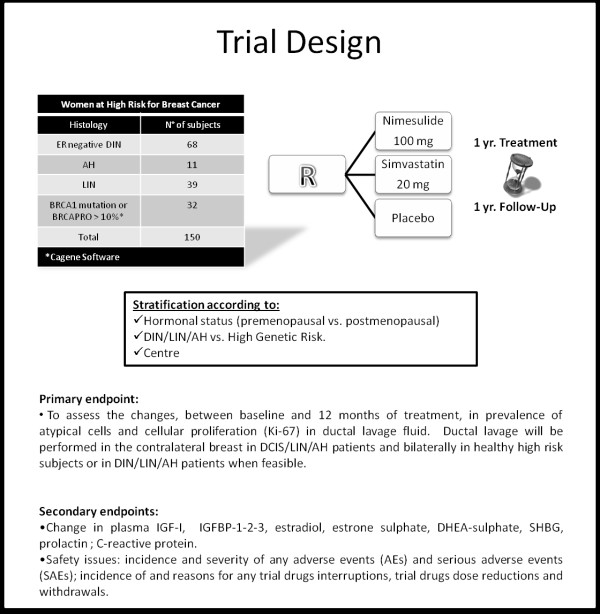
Trial design.

We are conducting a monoistitutional phase II, randomized, double blind, placebo controlled trial to assess the efficacy and the safety of a daily administration of nimesulide or simvastatin to change the expression of a large set of tissue and circulating surrogate endpoint biomarkers (SEBs) of breast carcinogenesis in women at higher risk of developing a hormone non-responsive (ER negative) breast cancer. The primary endpoint is the change in prevalence of atypical cells and cell proliferation (Ki-67), after 12 months of treatment. A total of 150 women were randomized: 50 per arm. Within the 3 treatment groups, subjects were stratified according to their hormonal status (premenopausal vs. postmenopausal ) and Ductal Intraepithelial Neoplasia (DIN)/Lobular Intraepithelial Neoplasia (LIN)/Atypical Hyperplasia(AH) vs. High genetic risk and centre. A schema of the trial design is presented in Figure 1.

### Participants selection

Eligible subjects are women at increased risk for hormone non-responsive BC: patients with previous surgery for ER negative Intra Epithelial Neoplasia (IEN), and unaffected subjects carrying a mutation of BRCA1 or with high probability of BRCA1/2 mutation. This represents a relatively heterogeneous group with various degrees of risk, but all characterized by a higher probability to develop a ER negative breast cancer rather than other cohorts of subjects. Their risk is often confirmed by a diagnosis of IEN at young age and/or by an early germ-line mutation assessment. Moreover, no current preventive treatments (especially endocrine) could be reasonably proposed to most of these subjects.

– Patients with ER negative DIN (within 12 months from radical surgery)

– Patients with AH (within 12 months from radical surgery)

– Patients with LIN (within 12 months from radical surgery)

– Unaffected carriers of BRCA1 mutation

– Unaffected subjects with high probability of BRCA1/2 gene mutation (≥ 10 % according to Berry Parmigiani and/or Couch model).

**Figure 2:**
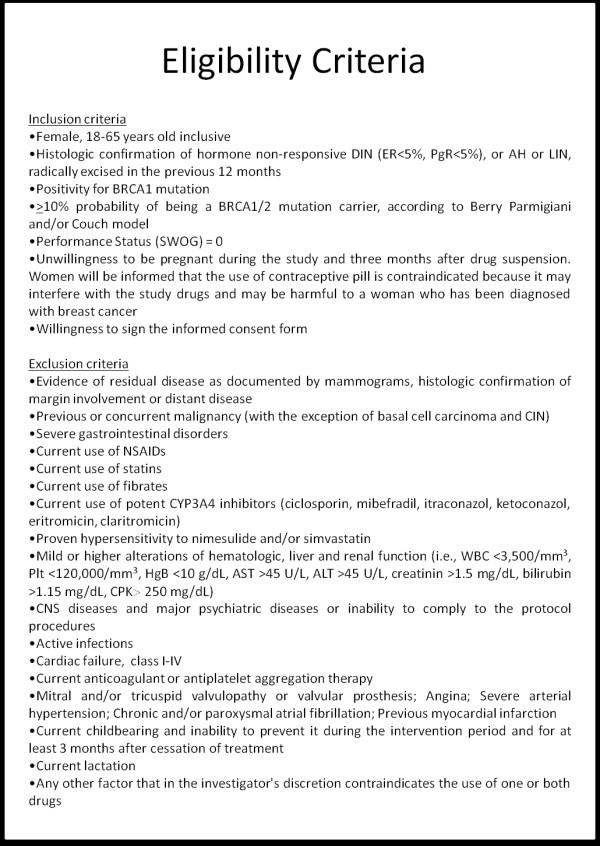
Inclusion and exclusion criteria.

On the basis of a weekly multidisciplinary meeting the candidates are contacted by phone by trained personnel illustrating the possibility of taking part in a chemoprevention trial and scheduling an appointment for an outpatient visit at the European Institute of Oncology. In case of a previous diagnosis of IEN, randomization is performed within 12 months from surgery. Inclusion and exclusion criteria are summarized in Figure 2.

### Recruitment and retention strategy

#### Pre-initiation phase

Subjects with a previous surgery of ER negative IEN, and unaffected subjects carrying a mutation of BRCA1 or with high probability of BRCA1/2 mutation that are potentially eligible for the trial are addressed to the oncologist and the geneticist respectively.

#### Active recruitment

After the evaluation by the oncologist of the multidisciplinary team, patients surgically treated for IEN receive a Hospital Discharge Report including an appointment to discuss the trial and, when possible, the alternative options. Unaffected subjects carrying a mutation of BRCA1 or with high probability of BRCA1/2 mutation on the basis of the geneticist evaluation are contacted by the staff of the Division of Cancer Prevention and Genetics for a first phone interview and the check of all inclusion criteria. The trial design is explained and willingness to participate is asked. Candidates accepting to participate or interested in the study are invited for a clinic visit at the European Institute of Oncology (EIO) or the Department of Oncology & Haematology of the University of Modena and Reggio Emilia for informed consent and baseline visit.

#### Retention phase (including adherence strategies)

Subjects are scheduled for periodic visits (every 3 months in the first year) during which a complete physical exam and lab tests are performed. A couple of weeks before the following scheduled appointment, participants are reminded about the visit, blood test and physical exam. Recruitment and retention effort are evaluated routinely by the site coordinator and the study staff.

#### Treatment groups

Patients who signed an informed consent and who met the eligibility criteria are randomly assigned to one of three groups for 12 months of treatment, as follows.

Group 1: nimesulide 100 mg/day, administered per os and on full stomach. A single oral dose of 100 mg of nimesulide suppresses COX-2 activity by 90% in both in vitro and ex vivo assays and, at a much lesser extent, COX-1 activity with a 20-fold selectivity for the former isoenzyme [23]. This dose is half the standard dose to obtain a faster effect on pain control and inflammation, but it may represent an active and safer dose for testing the chemopreventive efficacy of nimesulide. Moreover, this dose is able to reach a plasma concentration of 2–4 μg/ml [23], which is more than 10 times the IC_50_ necessary for the inhibition of COX-2 activity in blood assays, whereas it is five times lower than the IC_50_ for COX-1 inhibition [24]. Therefore, 100 mg/day appears a reasonable dose for chemopreventive purposes implying prolonged administration.

On May 15, 2007 the Irish Medicines Board (IMB) decided to suspend nimesulide from the Irish market and refer it to the EU Committee for Human Medicinal Products (CHMP) for a review of its benefit/risk profile. The decision was due to the reporting of six cases of potentially related liver failures to the IMB by the National Liver Transplant Unit, St Vincent Hospital. These cases occurred in the period from 1999 to 2006. On September 21, 2007 the EMA released a press release on their review on the liver-related safety of nimesulide. The EMA concluded that the benefits of these medicines outweigh their risks, but that there was a need to limit the duration of use to ensure the risk of patients developing liver problems is kept to a minimum. Therefore the EMA has limited the use of systemic formulations of nimesulide to 15 days. According to the European Institute of Oncology Ethics Committee communication, released officially on October 10, 2007 which recommended the maintenance of the study according to the present design, we are now performing an even more careful monitoring of the study participants and we are carrying out a systematic check of the possible side effects, both in those who are receiving treatment and in those who have finished. We have modified the Inform consent and we have informed all the participants accordingly.

Group 2: simvastatin 20 mg/day. The most important adverse events associated with statins are asymptomatic increases in liver transaminases, and myopathy. Myopathy and its serious complication, rhabdomyolysis, are potential side effects of therapy with the available statins, but occur very rarely. The molecular and biochemical mechanisms of myopathy and rhabdomyolysis caused by statins are yet to be fully elucidated [25]. However, a compilation of all randomized statin trials revealed that among 83,858 patients randomly assigned to receive either statin treatment or placebo, there were only 49 cases of myositis and 7 cases of rhabdomyolysis in the statin groups, compared with 44 cases of myositis and 5 cases of rhabdomyolysis in the placebo groups [26].

Group 3: placebo. An identical appearing tablet containing placebo is taken daily by participants assigned to the placebo group.

Toxicity is evaluated at each visit using the NCI toxicity criteria (CTCAE version 3.0, published 12/12/03). Any use of systemic drugs is clearly documented (time, doses, routes, and indications) and strictly followed by the physician. All medications (prescription and over-the counter), vitamin and mineral supplements, and/or herbs taken by the participant are documented on the concomitant medication CRF and included: start and stop date, dose and route of administration, and indication for use. Medications taken for a procedure (e.g., biopsy) are included. Patients are discouraged from taking unspecified medications.

### Adherence/compliance to treatment

Although drug concentration is not being measured in the blood, compliance is monitored in the following ways.

#### Patient self-report

Patient's history—the most direct source of information—is the most widely employed measure of a patient's adherence to their medical regimen. However, patient self-reporting has been criticized as being too subjective, with patients tending to over-report their adherence by as much as two to fourfold. Positive information is helpful, but false negatives are common.

#### Calendar completion

Each subject is given a 7-month calendar as a reminder of drug consumption. Each subject is asked to cross the corresponding day of the calendar (1 cross for each consumption). Subjects are asked to fill the calendar and some additional space is left for patient's notes. Each calendar is returned at the next scheduled visit.

#### Dose count

Each subject receives a 6-month supply (at baseline and 6th month visits) of the drug or placebo and they are asked to return all full and empty packs, irrespective of their use. Pills are counted and the compliance is measured as follows: number of pills taken (i.e., number of pills given-number of pills returned)/number of pills that should have been taken during that period of time. If packs are not returned, the compliance is calculated from the calendar. In case of discrepancy between the pill counts and the calendar, pill count is taken as the superseding compliance measure.

## Clinical evaluation and procedures

Date of birth, occupation, complete address and phone number, full details of family doctor; previous medical history; general physical examination, including anthropometric features; first or second degree family history of cancer; number of pack-years of cigarettes; alcohol consumption; concomitant medications.

Participants are assessed at baseline, 3, 6, 9, 12 and 24 months with clinical examination and blood safety tests. Mammography are performed at baseline, 12 and 24 months. DL are performed at baseline, 12 and 24 months in all subjects. In case of anatomical impediments to perform DL, this is substituted by a breast fine needle aspiration (FNA), preferably ultrasound-guided in the most dense area of the breast. Breast ultrasound is performed in premenopausal women at baseline, 12 and 24 months, in postmenopausal only if indicated by the study physician. Blood drawing for biomarkers and hormone measurements are done at baseline, 12 and 24 months. Intervention with nimesulide or simvastatin or placebo continues for 12 months or until the occurrence of a serious adverse event, including breast cancer. A detailed follow-up schema is illustrated in Table 1.

**Table 1:** Follow-up procedures and main outcome measures

		**Month**				
***Tests and Procedures***	***0***	***3***	***6***	***9***	***12***	***24***
Informed consent	X					
Eligibility checklist	X					
Medical history	X					
Mammography	X				X	X
Breast Ultrasound (if indicated)	X				X	X
Physical examination	X	X	X	X	X	X
Ductal Lavage (or FNA)	X				X	X
Concomitant medication	X	X	X	X	X	X
AE/ADR (*)	X	X	X	X	X	X
Hematology/biochemistry	X	X	X	X	X	X
Hormones, IGFs, SHBG, C-Reactive Protein	X				X	X

(*) *AE*/*ADR*, *adverse event*/*adverse drug reaction*.

### Methods for ductal lavage and fine needle aspiration

**Figure 3:**
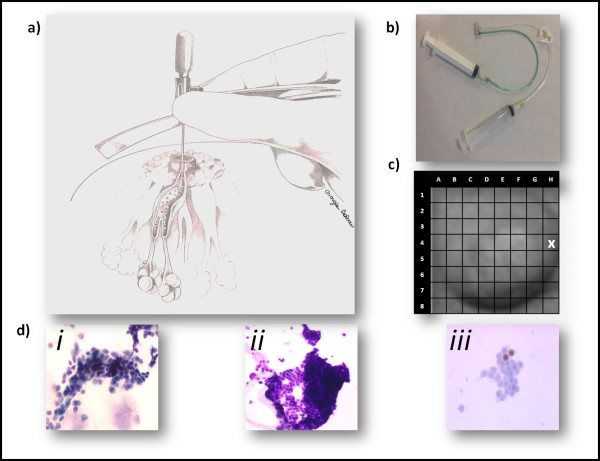
**Ductal Lavage. a |** saline is injected through the catheter into the duct and the breast is massaged to bring ductal cells into the chamber of the catheter. An empty syringe attached to the catheter is used to collect the cells from the catheter chamber. Saline injection and massage are repeated until a sufficient sample has been collected. **b** | microcatheter**. c** | nipple grid**. d** | Examples of ductal epithelial cells collected by ductal lavage: *i*) ductal hyperplasia; *ii*) atypical ductal hyperplasia; *iii*) ki-67 expression.

DL is performed by a single physician for both recruiting centers with a dedicated microcatheter (Sterylab, Rho, Italy) to cannulate ductal orifices on the nipple that are identified by nipple discharge. After the duct has been cannulated, and a small infusion of local anesthesia is performed, the milk duct is infused with a saline solution. DL is performed in the contralateral breast in DIN/LIN/AH patients and bilaterally in healthy high risk subjects or in DIN/LIN/AH patients when feasible. The fluid collected from the effluent tube is then analyzed for the presence of cell alterations. Ki-67 expression in epithelial cells obtained by DL is calculated using the percentage of cells expressing the antigen over the total number of epithelial cells. The localization of the cannulated duct is recorded on a specific nipple grid in order to collect samples always from the same breast duct (Figure 3).

In case of anatomical impediments to perform DL, this is substituted by a breast FNA, preferably ultrasound-guided in the most dense area of the breast. In all samples obtained by FNA, ER, PgR, and Ki-67 are analyzed using the immunohistochemical (IHC) method. Ki-67 is expressed as the actual percentage of stained cells over a total of at least 2000 tumor cells at high magnification (400 x).

### Methods for biomarker measurements

Fasting blood samples for circulating biomarkers are collected and stored at −80°C until assayed. All the circulating biomarkers are determined on serum. IGF-I and prolactin are measured by a chemiluminescent immunometric assay (Nichols Institute Diagnostics, San Juan, CA). The assays are performed on the automatic instrument LIAISON (DiaSorin Deutschland GmbH, Dietzenbach, Germany). IGFBP-1 is measured by a two-site immunoradiometric assay, IGFBP-2 by a double-antibody radioimmunoassay (RIA) and IGFBP-3 by enzyme-linked immunosorbent assay, all provided by Diagnostic Systems Laboratories Inc. (Webster, TX). IGF-I and IGFBP concentrations are expressed in nanomolar (nmol) concentrations to calculate the IGF-I/IGFBP ratio, which is used as a more sensitive index of growth factor bioavailability. SHBG and C-reactive protein are determined by a chemiluminescent immunometric assay provided by Diagnostic Products Corporation, Inc. (Los Angeles, CA) designed for the Immulite Automated analyzer. Estradiol is measured by a 3^rd^ generation RIA provided by Diagnostic System Laboratories Inc. (Webster, TX). Estrone-sulphate and Dehydroepiandrosterone-sulphate are determined by RIA kits provided by Diagnostic System Laboratories Inc. (Webster, TX). Pre- and post-treatment blood samples obtained from each subject are assayed within the same run to improve analytical precision. All analyses are blinded to the treatment groups.

## Toxicity and dose modification

Toxicity is evaluated at each visit using the NCI common terminology criteria for adverse events (CTCAE, version 3.0, revised June 10, 2003 http://ctep.cancer.gov). If grade 1 toxicity occurs, the participant is maintained on full doses. Toxicity is checked depending on clinical relevance but at least within 3 months by telephone or clinical visit. Grade 2 adverse events is monitored and treated depending on clinical relevance but at least within 3 months by clinical visit. If grade 2 toxicity should occur, treatment may not be stopped but reduced at 50% of the dose for 1 month and symptomatic relief is started. If grade 2 toxicity does not improve at all after 1 month at half dose (i.e. capsule taken on alternate days), treatment is stopped; otherwise it may be restored at full dose. For grade 3 or 4 toxicity, subjects come off study. Even after premature stopping of treatment, follow-up continues unmodified.

## Statistical consideration

### Sample size

A total of 150 subjects are allocated in the three treatment groups: 50 for each treatment group. Within the 3 treatment groups, subjects are stratified according to their hormonal status (premenopausal vs. postmenopausal), DIN/LIN/AH vs. Genetic High Risk, and centre.

Such sample size yields a power of 80% to detect a reduction from an anticipated 50% 12-month prevalence of atypical hyperplasia and cellular proliferation (Ki-67) in the control arm to 25% in each of the treated arms. The specification of an alpha level of 0.1 and a one-sided test were used in the calculation of the power. Both choices of an alpha level greater than 0.05 and a one-sided test are justifiable in phase II studies, also considering that the study is interested in determining if the prevalence of atypical hyperplasia and cellular proliferation (Ki-67) is reduced by the investigated treatments. The planned sample size incorporates a correction for possibly inadequate tissue sampling in about 20% of study subjects.

### Statistical methods

Each of the two active treatment groups will be compared separately with the control group for 12-month prevalence of atypical hyperplasia and cellular proliferation (Ki-67), considered as two distinct endpoints. The comparison will be based on Pearson’s Chi-square test. A secondary analysis will be carried out within a Generalized Estimating Equations (GEE) logistic regression modeling approach. Such analysis will incorporate baseline and 12-month measurements, and will take into account possible correlation between the two assessments made within the same subject. Secondary endpoints considered in this study are represented by continuous variables. Treatment effect on their change over time will be investigated by means of covariance analysis (ANCOVA), possibly after suitable data transformations to achieve normal distributions.

### Randomisation

A randomization list is prepared using permuted blocks to ensure that an equal number of subjects are assigned to the 3 arms at different points of the randomization process. Neither the investigators involved with the study nor the women participating know which type of preparation is administered. The treatment and pertinent arm to which a patient has been allocated is made known only in the case of proven need (e.g., severe adverse events) by the Data Center upon formal written authorization of the Principal Investigator.

### Blinding

All participants, those who administer the therapy and those who assess the outcomes are blinded to group assignment to ensure the double blind design. Both nimesulide and simvastatin have been purchased from the market, placebo capsules have been prepared by the Hospital Pharmacy of the European Institute of Oncology (EIO). All the treatments are blinded by over-encapsulation and appear as capsules (size 0) of the same colour and exactly identical. Drug or placebo are provided in boxes (of identical shape) containing the capsules needed for each semester. They are manufactured by the EIO pharmacy. The code will be revealed to researchers and participant once recruitment, data collection, and laboratory analyses are complete.

### Recruitment and preliminary results

**Figure 4:**
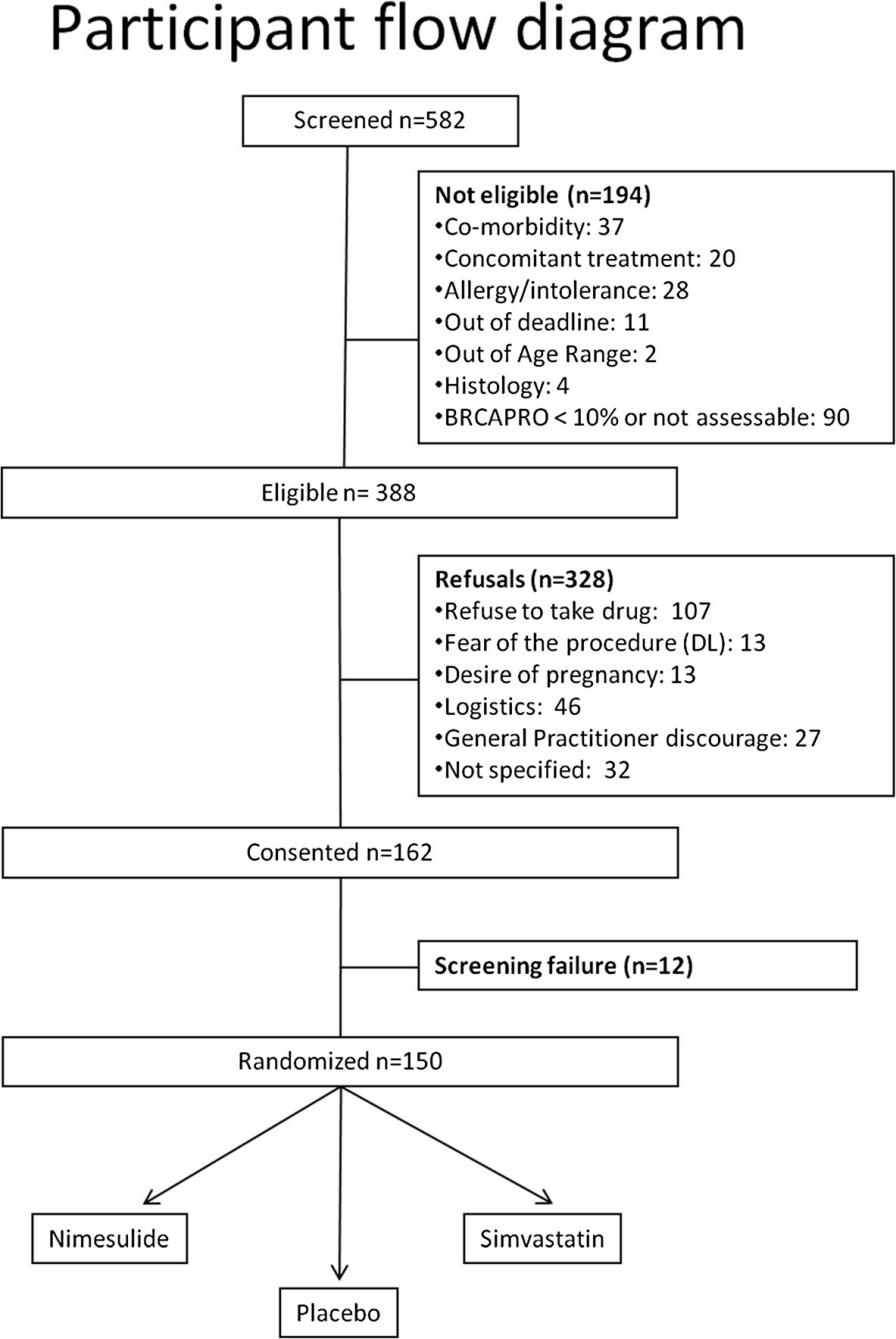
Number of subject for each phase of the randomized trial.

Among the 528 women evaluated for protocol inclusion, 388 were eligible according to inclusion criteria characteristics. The flow diagram of study of the randomized trial is shown in Figure 4. The accrual phase ended in 2011, with the randomization of the planned 150 subjects: 68 ER negative DIN, 50 LIN and AH and 32 subjects with BRCA1 mutation or high mutation probability. So far 125 women have completed the study. Ductal lavage has shown to be a feasible and reproducible procedure (only 7 patients who underwent DL at baseline shifted to FNA at 12 months). The treatment is very well tolerated and the safety blood tests did not show any significant liver toxicity, only few grade 1 for one of the liver enzymes. Only few subjects had CPK alteration with grade 1 toxicity. One case was included in the study with a baseline level of CPK grade 2 by mistake and the patient was withdrawn from the study. Including this last case, five subjects dropped out: two for gastrointestinal symptoms, one for muscle ache, and one refused to continue.

## Discussion

The success of chemopreventive approach depends on the recognition of high-risk subjects, the development of novel and safe agents, and the identification of new surrogate endpoint biomarkers using molecular pathways and new targets of drugs activity. Several chemoprevention studies have demonstrated that it is possible to reduce the incidence of hormone receptor positive breast cancer and that chemoprevention is clinically safe and well tolerated. Unfortunately we have no effective agents to prevent ER-negative breast cancer which accounts for 20–30% of breast cancers and has a poor prognosis [27]. Thus, it is worth identifying biomarkers and agents that are effective in the treatment and prevention of these subtypes. Several classes of new agents modulating the non-endocrine biochemical pathways have been developed and many of these are still currently under investigation. These agents include retinoids, epidermal growth factor receptor (EGFR), tyrosine kinase inhibitors (TKIs), cyclooxygenase-2 (COX-2) inhibitors, bisphosphonates, vitamin D receptor (VDR), statins, peroxisome proliferator activated receptor (PPAR), and others [28]. Safety is a major issue to take into account, since large randomized chemo-prevention trials have shown that few serious adverse events can prevent widespread public acceptance of preventive agents despite their proven efficacy. Our preliminary results showed that the treatment is very well tolerated and the safety blood tests did not show any significant liver toxicity. Ductal lavage has been proposed as a minimally-invasive, well-tolerated tool for obtaining breast epithelial cells for cytological evaluation of breast cancer risk. However, our trial might have some limitations. Ductal lavage can be highly time consuming, restricting its utility as a high-throughput clinical method. Furthermore, the effluent lavage fluid is highly diluted, thus potentially limiting its utility in possible future biochemical analysis. In spite of consistent data on Ki67 as a prognostic marker in early breast cancer, its role in atypical cells is uncertain. Furthermore, the variation in analytical practice markedly limits the value of Ki67 in this context. We assume to start the analysis of the primary and secondary endpoints within a year. To our knowledge, this is the first randomized placebo controlled trial to evaluate the role of ductal lavage to study surrogate endpoints biomarkers and the effects of these drugs on breast carcinogenesis.

## Competing interests

The authors declare that they have no financial or non-financial competing interests.

## Authors’ contributions

BB, AGG and DR have made substantial contributions to conception and design. ML, AGG, SM and DR have been involved in drafting the manuscript, analysis and interpretation of data. ML, DS, MC, LC have recruited participants and they will performed the follow-up at outpatient clinic. CC shall carry out pathological analysis. CJ shall prepare the drugs and placebo. MC is the physician who will perform ductal lavage. MP is the radiologist who will read the mammograms and who will perform breast fine needle aspiration. Statistical analysis will be performed by DR. All authors have read and approved the final manuscript.

## Pre-publication history

The pre-publication history for this paper can be accessed here:

http://www.biomedcentral.com/1471-2407/12/575/prepub
